# Ten simple rules for a successful EU Marie Skłodowska-Curie Actions Postdoctoral (MSCA) fellowship application

**DOI:** 10.1371/journal.pcbi.1010371

**Published:** 2022-08-18

**Authors:** Philipp Baumert, Francesco Cenni, Mikhail L. Antonkine

**Affiliations:** 1 Exercise Biology Group, Faculty of Sport and Health Sciences, Technical University of Munich, Munich, Germany; 2 Faculty of Sport and Health Sciences, University of Jyväskylä, Jyväskylä, Finland; 3 Unit of Health Research and Biotechnology, Bavarian Research Alliance GmbH, Munich & Nuremberg, Germany

## Introduction

The success of an academic career is dependent upon research accomplishments. The single most important factor for the success of a researcher on the academic job market is the scientific output, i.e., published papers [1], accompanied by other factors such as networking and participating in science communication. Academic mobility helps to create international collaborations and is seen very positively in job applications. Only 4% of researchers move to a new country, but mobile scholars have about 40% higher citation rates compared to non-mobile colleagues [2]. Further, researchers need to raise funding to successfully execute their own projects and to gain scientific independence. For this, postdocs and principal investigators need to know how to win grants independently [3,4].

The Marie Skłodowska-Curie Actions (MSCA) specifically support the aforementioned factors and help researchers to develop their skillsets. The MSCA is part of the ninth European research and innovation framework programme *Horizon Europe* and is the European Union’s (EU) flagship career development support programme with a budget totalling €6.6 billion from 2021 to 2027. The programme comprises a set of major research funding schemes including the renowned MSCA Postdoctoral Fellowships, Doctoral Networks, Staff Exchanges, Co-funding of regional, national, and international programmes (COFUND), and MSCA Citizens schemes.

The aim of MSCA Postdoctoral Fellowships are to enhance the creative and innovative potential of postdoctoral researchers with new skillsets acquired through advanced training as well as international, interdisciplinary, and intersectoral mobility. MSCA Postdoctoral Fellowships support researchers with a doctoral degree and a maximum of 8 years of post-graduate experience. The mobility of researchers is a key requirement in all MSCA schemes. There is a general rule that researchers cannot have resided or carried out their main activity in the country of the host institution for more than 12 months in the 36 months immediately prior to the call deadline. This requirement is intended to direct researchers holding a PhD to move abroad to work on cutting-edge research projects. In the new *Horizon Europe* framework programme, there are 2 types of MSCA Postdoctoral Fellowships. The *European Postdoctoral Fellowships* support a stay of up to 24 months in any European host institution. The *Global Postdoctoral Fellowships* include an “outgoing phase” of 12 to 24 months to a host institution in a non-European country, followed by a mandatory “return phase” of 12 months at a host institution based in an EU Member State or a *Horizon Europe* Associated Country. In the MSCA programme, a host institution can be a public (university or research institute) or a private (company, university, or research institute) entity. Further opportunities to review include MSCA COFUND actions. These are schemes of doctoral and post-doctoral research training programmes, which are partly funded by the EU/MSCA. These schemes have more tailored eligibility criteria and sometimes have smaller budgets. However, the application success rate of these MSCA COFUND actions is usually higher.

A successful application for the prestigious MSCA Postdoctoral Fellowship is associated with multiple positive long-term career developments, such as an improved likelihood of developing international collaborations, as well as producing more and better-cited publications in high impact journals. These are important stepping-stones for achieving principal investigator and professor positions, thus MSCA fellows are more successful at advanced career stages [5,6]. The positive effects on the career progress of the successful MSCA applicants can be partly attributed to the training aspects that go along with the MSCA Fellowship. Furthermore, reviewers of MSCA Fellowship applications are able to make a rough assessment of the most promising leading scientists in the next generation, who then predictably have successful scientific careers [5,7].

Despite the impressive total of 1,156 supported applicants in the year 2021 alone, the success rate of the MSCA Postdoctoral Fellowship programme is between 10% to 16% depending on the year and scientific area [8], highlighting its competitiveness. This paper shares 10 simple rules for preparation of a successful Postdoctoral Fellowship and COFUND application in the MSCA scheme. However, the application process for the other EU funding schemes is similar and much of our advice can be more broadly applied. Focusing on the MSCA Postdoctoral Fellowship application sets this paper apart from 2 other valuable contributions previously published in the “Ten simple rules…” series, as these publications shared more general guidance for (Postdoc fellowship) applications [9,10]. The team of authors is composed of recent MSCA postdoctoral fellows and reviewers who have evaluated MSCA applications for several years.

## Rule 1: Familiarise yourself with the MSCA funding programme and do not hesitate to ask for help

Make sure that you understand the overall logic as well as specific requirements of the MSCA programme. This helps you to develop your idea in the proper context and to successfully address both the call text as well as specific questions and points in the project template. Review the most up-to-date MSCA *Guide for Applicants*, which you can find on the European Commission website. There you can also find relevant guides for more specific issues (e.g., ethics). For MSCA, as well as for other programmes, the EU publishes the evaluation form well before the submission deadline. It can help you to better understand what is expected from your proposal and allow you to look at it from the reviewers’ perspective. If you have questions after reading these documents, do not hesitate to ask the persons in your institution responsible for EU funding or your national contact point (free of charge) for help and explanations. Further, free seminars and webinars that explain the current EU framework programme and the MSCA funding scheme are offered frequently from the regional to the EU level. Note that attending such an event in person presents you with an excellent networking opportunity. The better you understand the aim and the structure of the MSCA fellowship, the better you can design your application.

## Rule 2: Be sure to develop a competitive CV

The overall aim of your MSCA (or any other fellowship) application is to convince reviewers that you, along with your project idea, are worthy of being funded. Ideally, you have published one or more first author papers, showing a publication track record that is above average for your career stage and research field. However, the widespread belief that a successful MSCA applicant needs to have published a CNS (cell, nature, and science) paper is not correct [6]. There is also a vicious cycle of not getting grants unless you demonstrate that you are capable of winning funding [11]. To break the vicious cycle, it is helpful to apply for smaller grants first (e.g., travel grants, internal university grants, national fellowships). A proven grant history will help you to convince EU reviewers that you will be able to complete your project and you, together with your proposed project, are worthy of being funded.

## Rule 3: Develop your idea properly

Your idea is the heart of the entire process. It should be clear that you know the relevant literature comprehensively and which knowledge gap you plan to address. New ideas do not come fully formed, but are often developed and refined through a long process of reading, writing, and discussing with your peers. You can develop your research field by addressing a research question with a new state-of-the-art methodology. Conversely, you might be an expert on a particular methodology and you can use this tool for investigating a new research field. In both cases, an international expert in this methodology or research field could be your new host (see Rule 4). Choose a topic that you are passionate about, since in the best-case scenario, you will be successful and you will stay with it for several years. Take your time to develop your idea thoroughly, to make it clear and understandable, so that you feel comfortable to discuss it with colleagues and your future host. If the concept is not yet clear in your mind, how will you then be able to convince reviewers? Also, keep in mind the feasibility of realising your idea: The MSCA Postdoctoral Fellowship will support you for up to 2 years and the Global Fellowship for 3 years, so you need to have the required expertise (from you, from the host, and, if useful, also from external partners) and a reasonable time plan.

Try to put your research idea into a broader perspective. When thinking about your project, consider how it can contribute to tackling the challenges identified in *Horizon Europe* and the EU missions, as well as to achieving the United Nations Sustainable Development Goals. Do not be overwhelmed by this: Nobody expects you to solve these big problems, but rather to consider how your work could fit into the overall strategy of addressing them. This will greatly help you in writing the “Impact” section of your MSCA application later (see Rule 7). The amount of time required for preparing your application is clearly subjective, but according to our experiences, you should expect to spend at least 2 full months doing so, including the reviewing process of others.

## Rule 4: Find a good match with the host institution

Start early with finding your host lab and host organisation. Of course, you should look carefully at the track record of your future supervisor, especially with regard to scientific publications, experience in training PhD students and postdocs, success with funding acquisition, and overall academic reputation. Also, choosing international universities that are rated as excellent will increase your chance of receiving funding [12]. It is crucial to find an academically strong host that has expertise complementary to yours, with people, resources, and facilities suitable for implementing your project idea. Note that these points are not only important for achieving the aims of your project, but are also important questions during evaluation of your application (see also Rule 5). Make sure your future host is truly interested in supporting you towards your objectives. You should expect them to be actively supporting you during the application process as well as during your fellowship. The perfect scenario is to spend some time at the host institution beforehand (e.g., supported by a small visiting fellowship) and get to know the people and the environment, thus showing that the research bond is already established. A red flag is a potential host who does not respond regularly. This is not only painful during the application process, but they will probably continue to behave in a similar way during the project. Your most important goal is to find a host who will support you well beyond your time as MSCA fellow, i.e., in your academic future, especially when you apply for more advanced positions later.

## Rule 5: Highlight the 2-way transfer of knowledge

Part of evaluation criteria “Excellence” (see Rule 7) in MSCA Postdoctoral Fellowships is ensuring 2-way (3-way in case of Global Fellowships) knowledge transfer. While choosing your host, keep this criterion in mind. On the one hand, discuss with your potential host which crucial skills you are currently missing and could gain if you join their team with the MSCA Postdoctoral Fellowship. On the other hand, it is also necessary to discuss the research skills that are currently missing in their team but you could contribute. It is actually important to identify and clearly describe such knowledge gaps, thus making the 2-way knowledge transfer in your application more convincing.

In addition, if you identify a knowledge gap in your CV or proposal and the host cannot fill it, you have the option to plan a secondment or placement in a nonacademic sector. MSCA researchers may be seconded to other institutions for up to one-third of the fellowship duration (one-third of the outgoing phase in case of Global Fellowships). Such a secondment allows you to improve your training by learning new skills and gaining new knowledge. Note that knowledge and skills you learn during a secondment could also be part of your contribution to the knowledge transfer to your host. At the end of the fellowship, an MSCA researcher may also apply for a placement (up to 6 months) in a nonacademic sector in Europe (EU Member State or *Horizon Europe* Associated Country) to complement their academic training. When choosing a host for secondment and placement in the nonacademic sector, use the same rules as when choosing your future host institution (see Rule 4). In your application, do not forget to provide convincing arguments for the necessity of the secondment and the placement in a nonacademic sector. The logic of your choice should be sound and clearly explained.

## Rule 6: Study and strictly follow EU proposal template

The European Research Executive Agency made an effort to create specific proposal templates for the MSCA Postdoctoral Fellowship and as well as for other EU funding programmes. Each template contains several points that the applicant is expected to address. Make sure that you understand everything on the proposal template—otherwise look for help (see Rule 1). As EU proposal templates are updated on a regular basis, be sure you have downloaded the most up-to-date version of the proposal template and use it to prepare your application before you start writing. Use both the structure as well as the numbering of the EU template to develop your application. Of course, you are free to change the numbering and choose an entirely different structure for your application. However, reviewers receive a document from the Research Executive Agency, where it is indicated in which parts of application certain points can be expected to be addressed. Why make their life more difficult?

We recommend seeking out successful MSCA Postdoctoral Fellowship applications from previous years. This will give you additional insights into the overall structure, necessary quality of presentation and depth of details, as well as the balance between the different parts of proposal. However, successful grant proposals are sometimes difficult to get. Ask colleagues who were successful with an MSCA application if they can share their proposal. If you do not know any successful MSCA applicants personally, look up past successful applicants in your research field or from your current or future host university, and ask them for help. You will not believe how many researchers will come back to you and share their knowledge together with their former proposals. Successful candidates have often been supported by previous successful candidates, and they are willing to repay the kindness to the next generation (“pay it forward” principle).

## Rule 7: Take care with all sections

There are 3 funding award criteria in EU’s research and innovation framework programme *Horizon Europe*: “Excellence,” “Impact,” and “Quality and Efficiency of the Implementation.” The “Excellence” section is the core of your project, where you explain the state of the art in your domain of science, your vision, the specific objectives of your project, and the methodology you will employ to achieve them. In “Impact,” you need to show what benefits your project can bring to the scientific and not-scientific communities, as well as how it will decisively advance your scientific career. In “Quality and Efficiency of the Implementation,” you should provide a convincing work plan for your project and explain how you can mitigate risks arising during the project implementation.

“Excellence” is the most exciting section and, quite naturally, researchers tend to focus on this section. Often it is truly outstanding—at the expense of other 2 sections of the application. However, you will not be successful if you have not spent enough time and effort on the other 2 sections. This can make the difference between great project idea and a funded project. Many researchers tend to forget this. Plan to invest a significant proportion of your time into the development of the dissemination part of “Impact” and how your research might influence other stakeholders, as well as working out details of the implementation strategy of your proposal.

The applicant is free to choose the allocation of the maximum of 10 pages between these 3 sections. However, it makes sense to approximately align the section lengths to the weighting of evaluation criteria of MSCA applications. The section “Excellence” is weighted 50%, “Impact” is 30%, and “Quality and Efficiency of the Implementation” is 20%. The rule of thumb is not to exceed 6 pages with the “Excellence” section. You need to get the maximal or near the maximal score for each of the 3 evaluation criteria in order to be successful.

## Rule 8: Proposal: Structure, structure, structure

Reviewers are busy people and reviewing is something that they do in addition to their everyday work. Therefore, you want to make their life as easy as possible. In additional to Rule 6, you have to structure your proposal as best as you can. Having a clear and comprehensive structure makes your proposal appealing, easy to read, and clearly understandable. Nobody will fund a project that they do not fully understand. Your application should be well written. It does not have to be perfect, but it is painful to have simple grammar mistakes and gives reviewer an impression that you did not care about the proposal.

How can you best convince reviewer that your project is well thought through? Each project has to have a comprehensive work plan. Divide your work into logical parts; these would be your work packages. For each work package formulate goals, tasks, and deliverables. Make sure that you understand the difference between a milestone, which is a decision-making point, and a deliverable, which is a tangible result of your work. A milestone is a control point for the entire project and could be connected to one or several work packages. A deliverable belongs to specific work package and usually corresponds to a specific task. Make sure that all your milestones and deliverables are formulated according to the above principles. Do not forget that administrative, i.e., project management, dissemination, and teaching tasks should also be reflected in one or several work packages and should have their own milestones and deliverables. A well-developed Gantt chart, which displays the proposed timeline of all work packages and tasks as well as the timing of milestones and deliverables, is absolutely essential. Think about how your work packages interact with and depend upon each other. For this, you can include a small diagram (PERT chart) illustrating these interactions. Make sure that you have identified real risks of implementation for your project, evaluated their likelihood and potential impact, as well as described convincing mitigation measures. Have in mind: If the proposed MSCA project has no risks, it is not a front-line research project and it will not be funded.

In contrast to some other funding schemes, ethical approval is not necessary for the actual submission of the MSCA application. However, the proposal should be “ethics ready.” In Part A, you need to fill out the Ethics Table. If you have answered “YES” to any of the questions there, then potential ethical concerns need to be addressed in the “ethics self-assessment” form as well as by providing additional documents if necessary. The guide *EU Grant How to complete your ethics self-assessment* leads you nicely through the “ethics self-assessment.” Be aware that specific research directions will not be funded by the MSCA programme, such as human cloning for reproductive purposes. Positively evaluated projects will undergo an ethical review process. In the past years, about 42% of proposals received ethics clearance [13]. Other positively evaluated proposals received conditional ethics clearance. Most of the identified issues were related to protection of personal data and environment/health and safety. Only in rare cases were proposals were rejected on ethics grounds [14].

## Rule 9: Get as much feedbacks as possible

Have you written your first draft? Great! Your first and most important goal is that the reviewers understand and appreciate your application. The best way to achieve this is to get other people to read your proposal and to creatively process their feedback. When it is ready, get advice from supervisors and colleagues on different parts as well as on the whole of your application. The “Excellence” section can be adequately reviewed by your research colleagues. However, not all of your “test readers” should be familiar with your research. We recommend that someone who is specialised in another field of science should also read the “Excellence” section of your application. If your idea is well described, they will be able to understand key points and the overall concept, and they will be excited about it. If not, then you should rewrite this section. Note that among the reviewers, there will almost certainly be a person who is not specialised in your research field.

There are usually experts for the sections “Impact” and “Quality and Efficiency of the Implementation” within your university or research institute who can help you improve their quality. Although not always applicable, an efficient solution is to prepare different parts of your proposal at different time points. This way you can send 1 section to your chosen person and, in the meantime, you can carry on with preparation of the other sections. Give realistic deadlines to each “test reader” and make sure to plan enough extra time to work on their feedback. Critiques from others are extremely valuable for you during preparation of your application, but you have to learn to be a selective listener. In the end, it is your idea and your application, so you have to make a final call on which suggestions you will implement in the proposal. Do not waste your time on unproductive generic advice. Talk to your mentor and to someone independent who frequently acts as reviewer. Ask them, what they usually look for, like, and dislike in funding applications. What are typical weak spots, which you also can look for in your application?

## Rule 10: Do not forget the final check of your application

You have likely already spent a couple of months thinking, reading, planning, and writing your application. Now it is time for reflection and the final check. Take a step back and look critically at important aspects of your application. Try to concentrate on things that often escape the attention of an applicant. For example, are your figures clear enough? Are the management of research data and open science practices addressed adequately? Have you identified and described the regular scientific and soft-skills courses that you want to take during the fellowship? Is the 2-way knowledge transfer between you and the host clearly recognisable in the application? Do you sufficiently address specific science communication, dissemination (especially towards the general public), and exploitation of your project results? Have you described in detail the handling of Intellectual Property Rights in your application? Check your milestones and deliverables. Make sure that they are correctly formulated and that not all of them are timed to the second half of the project. Does your Gantt chart include all the necessary information (see Rule 8)? Have you carefully considered potential project implementation risks and how you will deal with them?

## Conclusions

Each project idea develops according to its own dynamic and specifics of your research field. For example, your research partners might have specific wishes as well as other priorities in the project design. You have to consider these and other, sometimes unforeseen, circumstances, while developing your own research idea. With our 10 simple rules, we aim to give young researchers “food for thought” and a “push in the right direction” in the development of their project idea.

Have you been successful with your MSCA grant application? Congratulation, you are one of the few applicants who have achieved this career milestone. It is celebration time and you should enjoy it! Do you remember the “pay it forward” principle in Rule 6? It is now your turn to support future MSCA applicants. Be kind to specific requests, take your time with the response, and share your knowledge and experience generously.
